# Improved sleep quality is independently associated with decision-making recovery in panic disorder: a longitudinal study

**DOI:** 10.1038/s41598-026-37946-5

**Published:** 2026-02-04

**Authors:** Hediye Hilal Okucu, Deniz Alçı

**Affiliations:** 1 Psychiatrist Ermenek State Hospital Karaman, Ermenek, Turkey; 2 School of Medicine, Department of Psychiatry Cagis Campus, Balıkesir University, Balıkesir, 10145 Turkey

**Keywords:** Anxiety, Sleep disorders

## Abstract

Cognitive impairments are frequently observed in patients with panic disorder (PD), yet the relationship between sleep quality and cognitive recovery remains underexplored. This study investigated whether improvements in sleep quality during routine psychiatric care are prospectively associated with changes in decision-making in PD. Eighty-one patients with PD and 81 healthy controls were assessed using standardized clinical and cognitive measures, including the Pittsburgh Sleep Quality Index and Iowa Gambling Task. After three months of naturalistic follow-up, 38 patients were reassessed. Patients with PD initially exhibited significantly poorer sleep quality, more severe symptoms, and greater cognitive impairment than controls. Over the follow-up period, both clinical symptoms and sleep quality improved. Notably, improvements in sleep quality were independently associated with better performance on the Iowa Gambling Task, suggesting a relationship with decision-making performance. Changes in executive function showed only a non-significant trend in relation to sleep improvement. These findings suggest that improved sleep quality is independently associated with cognitive improvements, particularly decision-making, in PD. Addressing sleep disturbances in clinical care may be crucial for optimizing cognitive outcomes in patients with panic disorder.

**Supplementary Information:**

The online version contains supplementary material available at 10.1038/s41598-026-37946-5.

## Introduction

Panic disorder (PD) is characterized by recurrent and unexpected panic attacks. Although sleep disturbances are not included in the DSM-5 diagnostic criteria, recent studies have shown that many individuals with PD experience significant insomnia and poor sleep quality, which can severely impact daily functioning^1,2^. Approximately half of individuals with PD report clinically severe insomnia, which can impair attention, emotional regulation, and overall quality of life^2^. Therefore, even though sleep issues are not a diagnostic feature in PD, they remain a common and clinically relevant concern^3^.

Cognitive functions such as memory consolidation, executive functioning, and decision-making are strongly associated with sleep quality. Restorative sleep has been associated with better learning and memory, whereas inadequate or disrupted sleep has been linked to poorer attention, working memory, cognitive flexibility, and reasoning^4,5^. People experiencing sleep disturbances have been reported to show difficulties in risk evaluation and decision-making. Neuroimaging and behavioral research has demonstrated that sleep loss impairs prefrontal cortex-dependent functions, including emotional regulation^6,7^. In anxiety disorders, poor sleep is frequently reported and is associated with impairments in memory and executive functioning^8,9^. These findings suggest that sleep disturbances may be related to cognitive impairments by disrupting attentional control, working memory, and cognitive flexibility^10^.

Executive functions encompass a set of higher-order cognitive skills, including working memory, planning, cognitive flexibility, problem-solving, and decision-making^11^. Research into executive function in PD has been limited, as most studies focus on panic symptoms rather than cognitive processes^12^. However, tools such as the Wisconsin Card Sorting Test (WCST) and Iowa Gambling Task (IGT) are well-established methods for assessing cognitive impairments in PD^13,14^. While global executive dysfunction is not always present in PD, specific impairments in domains such as decision-making and cognitive flexibility appear to be more pronounced^12^. Even mild impairments in these cognitive domains can have a substantial impact on the daily functioning, occupational performance, and social interactions of patients^15^. Despite this, treatment approaches for PD often prioritize panic symptom reduction and neglect cognitive aspects^16^.

Importantly, improvement in panic symptoms does not necessarily imply recovery of cognitive functioning. Many patients continue to experience attention, memory, and decision-making difficulties even after achieving symptomatic remission^17^. This phenomenon is well-established in major depressive disorder, where cognitive impairments often persist beyond mood symptom resolution and hinder functional recovery^18^. Similarly, studies have shown that cognitive dysfunction in PD—such as impaired habituation and prepulse inhibition—can persist independently of panic symptoms^17,19^. In such cases, symptom-focused treatment may be insufficient, and cognitive functioning should be addressed as a distinct therapeutic target^20^.

To our knowledge, no longitudinal studies have specifically investigated whether spontaneous improvements in sleep quality—without targeted sleep interventions—contribute to cognitive recovery in patients with PD. This represents a clear gap in the literature. The present study aimed to evaluate whether changes in sleep quality over a three-month naturalistic follow-up period are associated with improvements in executive functioning and decision-making. By focusing on naturally occurring changes in sleep during standard treatment, this study offers a novel perspective on the link between sleep and cognition in PD, with implications for developing more holistic treatment strategies.

## Materials and methods

### Participants and procedure

The study included volunteer patients between the ages of 18 and 65 who were diagnosed with panic disorder (PD) based on DSM-5 criteria and presented to the Balıkesir University Faculty of Medicine psychiatry outpatient. Eligible participants had not taken psychotropic medication in the previous month and were physically and cognitively capable of completing the assessments. The required sample size was calculated using G*Power 3.1 (effect size = 0.5; α = 0.05; power = 80%), resulting in a minimum of 64 individuals per group. To increase statistical power and account for possible attrition, 81 patients were included in each group.

Diagnoses were confirmed through structured interviews conducted by a psychiatrist using the SCID-5-CV (Structured Clinical Interview for DSM-5). At baseline, participants completed the Sociodemographic Data Form, PSQI, PDSS, WCST, and IGT. The patient group was followed for three months with routine clinical care, and all scales were re-administered at follow-up. No additional interventions were implemented for research purposes. All participants were screened for primary sleep disorders through a structured clinical interview, including questions about snoring, witnessed apnoeas, restless legs, and other sleep-related complaints. In the patient group, two individuals had a previously diagnosed and medically managed primary sleep disorder (one obstructive sleep apnoea and one restless legs syndrome). Both were clinically stable at the time of the study and were retained in the main analyses; however, sensitivity analyses excluding these cases yielded virtually identical results. Family psychiatric history was assessed through patient interview and referred to the presence of a documented psychiatric diagnosis in first-degree relatives (parents or siblings), including mood disorders, anxiety disorders, psychotic disorders, or substance use disorders.

The control group consisted of hospital employees and their first-degree relatives with no psychiatric history, matched to the patient group by age, gender, and educational level. Among the 78 control participants, 19 (24.4%) were hospital employees who reported occasional night duties, whereas the remaining controls did not work night shifts. For the vast majority of these individuals, night shifts occurred no more than once per month, and only a few reported up to two night shifts per month. These duties generally allowed at least partial sleep and did not systematically lead to chronic sleep deprivation. In addition, a sensitivity analysis excluding all night-shift workers from the control group confirmed that the large group differences in sleep quality remained virtually unchanged (see Results and Limitations). SCID-5-CV was also used to confirm the absence of psychiatric diagnoses in this group. This sampling method was chosen due to limited access to a broader community sample.

Ethical approval was obtained from the Balıkesir University Non-Interventional Clinical Research Ethics Committee (Decision No: 2023/75, Date: 15.08.2023). Written informed consent was obtained from all participants prior to enrollment. All procedures involving human participants were conducted in strict accordance with the ethical standards outlined in the 1964 Declaration of Helsinki and its later amendments, the ethical guidelines and research regulations specified by the Turkish Ministry of Health, and the Balıkesir University Faculty of Medicine’s Ethics Committee guidelines for clinical research involving human participants. Additionally, diagnostic evaluations were performed using validated clinical instruments and structured diagnostic interviews based on Diagnostic and Statistical Manual of Mental Disorders, Fifth Edition (DSM-5) criteria. Confidentiality and privacy of all participants were strictly maintained, and all data were anonymized and securely stored in accordance with applicable national and international regulations.

### Measures and neuropsychological assessments

#### Panic disorder severity scale (PDSS)

The PDSS was created to evaluate the severity of PD symptoms and to monitor the treatment process. The Turkish validity and reliability study on the scale developed by Shear and colleagues was conducted by Monkul et al. In 2004^21,22^. This scale evaluates the frequency, intensity, and effect on the patient’s quality of life regarding panic attacks encountered in the past week. PDSS is extensively utilized in the clinical monitoring of PD in worldwide literature and is recognized as a dependable instrument for assessing therapy efficacy.

#### Montgomery–Åsberg depression rating scale (MADRS)

MADRS is a structured interview scale developed to measure the severity of depression symptoms, administered by physicians. The scale comprises 10 items and evaluates the emotional and physical aspects of depression. The Turkish adaption was performed by Kara Özer et colleagues. The study utilized it to identify and differentiate the associated depressive symptoms commonly observed in PD patients^23,24^.

#### Pittsburgh sleep quality ındex (PSQI)

The Pittsburgh Sleep Quality Index (PSQI) is a widely recognized self-report instrument that assesses individuals’ sleep quality over the preceding month. It encompasses seven sub-dimensions, including sleep latency, duration, and factors contributing to deterioration. The Turkish validity study on the scale created by Buysse et al. was conducted by Ağargün et al. (1996)^25,26^. It was utilized to objectively evaluate the sleep disturbances commonly seen in PD and to examine the impact of variations in sleep quality on cognitive skills.

#### Wisconsin card sorting test (WCST)

The WCST is a neuropsychological assessment that measures an individual’s executive functions, including cognitive flexibility, abstract reasoning, sustained attention, and problem-solving abilities. The assessment created by David A. Grant and Esta A. Berg was adapted into Turkish by Karakaş et al.^27,28^. This study utilized the computerized version. Participants were asked to match cards based on color, shape, or number. The matching rule changed periodically to assess cognitive flexibility and adaptability. WCST is a fundamental assessment in the study, designed to objectively evaluate impairments in executive function and to elucidate the impact of variations in sleep quality on these functions.

#### Iowa gambling task (IGT)

The IGT is a neuropsychological assessment designed to evaluate decision-making abilities under uncertain conditions. The test was created by Bechara and associates. The adaption study of the IGT was performed by Güleç and associates. Participants in the test are given 2000 Turkish Lira (TL) of virtual currency and must select from four decks, A, B, C, and D, with the objective of maximizing profit. The individual’s propensity for making risky choices is quantitatively assessed via favorable and disadvantageous decks. The IGT was used to assess decision-making deficits in patients with PD and to explore how these might be influenced by sleep quality. The computerized test was administered, and the net score was computed using the formula ([C’+D’]-[A’+B’])^29,30^.

All clinical and neuropsychological assessments were selected for their validated psychometric properties and relevance to PD-related cognitive deficits. The WCST and IGT were specifically chosen to evaluate executive function and decision-making, domains frequently reported as impaired in PD. Both the WCST and IGT are well-established neuropsychological instruments with extensive normative data in healthy adult populations. The WCST has demonstrated strong reliability and validity for assessing set-shifting, cognitive flexibility, and abstract reasoning, with normative performance patterns widely reported across different age and educational groups. The IGT has similarly been validated as a measure of affective decision-making and reward–punishment sensitivity, with normative samples consistently showing the characteristic shift toward advantageous decks over time. Performance in the present study was interpreted in relation to these normative patterns described in the literature rather than against a separate normative dataset.

All questionnaires used in this study are non-commercial research instruments and were used without additional permission where publicly available. Permission to use the validated Turkish version of the Iowa Gambling Task (IGT) was obtained from the developer of the adaptation. The Wisconsin Card Sorting Test (WCST) was administered using a research-based computerized format following the standard task structure described in the literature, without any licensed or proprietary commercial software.

### Treatment protocol

Patients received treatment in accordance with routine clinical guidelines at the psychiatry outpatient clinic. This typically included first-line pharmacological therapies such as selective serotonin reuptake inhibitors (SSRIs) or serotonin-norepinephrine reuptake inhibitors (SNRIs), adjusted according to symptom severity, comorbidities, and treatment history. Short-term anxiolytics and psychoeducation were used when clinically indicated. No experimental interventions were introduced for research purposes, and all care occurred within standard clinical practice. In this naturalistic longitudinal design, all patients continued to receive routine clinical care without any standardization imposed by the study protocol. Routine care typically consisted of follow-up visits every 4–6 weeks, psychoeducation about panic disorder symptoms, and pharmacological treatment when clinically indicated (usually SSRIs/SNRIs, and short-term benzodiazepines when required). No structured psychotherapy or additional intervention was provided. These procedures reflect evidence-based outpatient practice but were not controlled or manipulated by the research team.

All diagnostic and therapeutic interventions were performed in accordance with internationally accepted psychiatric treatment guidelines, including those of the American Psychiatric Association (APA), the World Federation of Societies of Biological Psychiatry (WFSBP), and the Psychiatric Association of Turkey^31–33^. All aspects of patient management adhered to current good clinical practice and ethical standards. Throughout the study, patient confidentiality and privacy were strictly maintained; all data were anonymized and securely stored to protect participant identities. Psychotropic medication use was systematically recorded using a structured case report form. At baseline, 79 out of 81 patients with panic disorder (97.5%) were receiving antidepressant treatment, in line with guideline-based first-line therapy. Benzodiazepines were prescribed to 12 patients (14.8%), typically on an intermittent basis for acute anxiety relief rather than as long-term hypnotic agents. Z-drugs were used by 2 patients (2.5%). Sedative antidepressants (e.g., mirtazapine, trazodone) were prescribed in 15 cases (18.5%), and other sedative agents in 3 cases (3.7%). Overall, 24 patients (29.6%) were classified as receiving at least one sedative medication. Information on dose adjustments, switches between antidepressants, and addition or discontinuation of benzodiazepines or sedative antidepressants was documented as part of routine clinical care. Medication decisions and adjustments were made solely on clinical grounds by the treating psychiatrists and were not dictated by the study protocol.

### Statistical analysis method

All statistical analyses were conducted using IBM SPSS Statistics 23.0. The distribution of quantitative variables was assessed using the Shapiro-Wilk test, Q-Q plots, and histograms. Since the data did not meet normality assumptions, non-parametric tests were used.

Descriptive statistics included mean, standard deviation, median, frequency, and percentage. The Mann-Whitney U test was used for between-group comparisons. Within-group changes from baseline to follow-up were assessed using the Wilcoxon Signed-Rank Test. Correlations between variables were examined using Spearman’s rank-order correlation.

To analyze repeated measurements (e.g., sleep quality, panic severity, and cognitive scores), Generalized Estimating Equations (GEE) were applied. GEE was selected for its ability to model correlated data and handle missing values effectively. In addition, a sensitivity analysis was conducted in which hospital employees with reported night duties (*n* = 19) were excluded from the control group to examine the robustness of between-group differences in sleep and cognitive measures. Categorical variables were analyzed using Pearson’s Chi-Square or Yates’ continuity correction when appropriate. Statistical significance was set at *p* < 0.05.

## Results

### Sample characteristics

The study included 162 participants, evenly divided between individuals diagnosed with PD (*n* = 81) and healthy controls (*n* = 81). The mean age of the participants was 35.64 ± 12.68 years. Of the total sample, 55.6% were female and 44.4% were male. There were no significant differences between the groups in terms of age, gender, or educational level. However, occupational status differed significantly between groups (*p* = 0.003) (see Table 1).

Additionally, clinical variables such as psychiatric history, family history of psychiatric illness, presence of physical illness, and current medication use were significantly different between patients and controls (see Table 1).

**Table 1.** Sociodemographic characteristics of the patient and control groups.

Section A. Sociodemographic Characteristics				
	Patient (n = 81)	Control (n = 81)	Test Value	p-value
Gender				
Male	35 (43.2%)	37 (45.7%)	10.100	0.752
Female	46 (56.8%)	44 (54.3%)		
Age (years)				
Min-Max Mean ± SD	18–65	18–65	2–0.699	0.485
	34.94 ± 13.08	36.33 ± 12.30		
Age group				
< 35 years	48 (59.3%)	43 (53.1%)	10.627	0.429
≥ 35 years	33 (40.7%)	38 (46.9%)		
Education level				
Primary school Middle school	11 (13.6%)	6 (7.4%)	11.920	0.589
	11 (13.6%)	10 (12.3%)		
High school	29 (35.8%)	30 (37.0%)		
University	30 (37.0%)	35 (43.2%)		0.635
Marital status				
Single	37 (45.7%)	34 (42.0%)	10.226	
Married	44 (54.3%)	47 (58.0%)		
Occupation				
Unemployed Housewife Civil servant	13 (16.9%)	10 (12.3%)	117.666	0.003**
	12 (15.6%)	3 (3.7%)		
	17 (22.1%)	40 (49.4%)		
Worker Self-employed Retired	6 (7.8%)	9 (11.1%)		
	23 (29.9%)	15 (18.5%)		
	6 (7.8%)	4 (4.9%)		

Section B. Clinical Characteristics				
	Patient (n = 81)	Control (n = 81)	Test Value	p-value
Psychiatric history				
Present	62 (76.5)	7 (8.6)	**76.367**	**<** 0.001**
Absent	19 (23.5)	74 (91.4)		
Psychiatric history of family				
Present	15 (18.5)	2 (2.5)	**9.464**	0.002**
Absent	66 (81.5)	79 (97.5)		
Physical illness				
Present	30 (37)	6 (7.4)	**18.893**	0.001**
Absent	51 (63)	75 (92.6)		
Medication use				
Present	18 (22.2)	3 (3.7)	**10.723**	0.001**
Absent	63 (77.8)	78 (96.3)		

Clinical characteristics		Patient (n = 81)	Control (n = 81)	Test Value	p-value
Psychiatric history	Present	62 (76,5)	7 (8,6)	**76,367**	**<** 0,001**
	Absent	19 (23,5)	74 (91,4)		
Family history of psychiatric disorders	Present	15 (18,5)	2 (2,5)	**9,464**	0,002**
	Absent	66 (81,5)	79 (97,5)		
Physical illness	Present	30 (37)	6 (7,4)	**18,893**	0,001**
	Absent	51 (63)	75 (92,6)		
Medication use	Present	18 (22,2)	3 (3,7)	**10,723**	0,001**
	Absent	63 (77,8)	78 (96,3)		

Qualitative variables are expressed as numbers (percentage), and quantitative variables as minimum–maximum and mean ± standard deviation. Statistical analyses were conducted using Pearson’s Chi-Square, Student’s t-test, Yates’ corrected Chi-Square, and Fisher’s Exact Test, as appropriate. A p-value of < 0.01 was considered statistically significant. Family psychiatric history = presence of a psychiatric diagnosis in first-degree relatives (parents or siblings).

Table 1. Sociodemographic and Clinical Characteristics of Participants.

### Group differences in clinical variables

The clinical scale scores in the PD group were significantly higher than those in the control group (see Tables 2, 3 and 4). The mean MADRS score was 12.16 ± 6.05 in the PD group compared to 2.21 ± 1.67 in the control group. The mean PDSS score was 18.44 ± 5.02 (control: 0.21 ± 0.47), and the mean PSQI score was 10.08 ± 3.07 (control: 4.14 ± 2.57). All between-group differences were statistically significant (*p* < 0.001).

**Table 2:** Evaluation of Pittsburgh sleep quality index (PSQI) scores in the patient and control groups.

	Patient (*n* = 81)	Control (*n* = 81)	Test Value	p-value
Min–Max	2–37	0–5	**124.50**	< 0.001**
Mean ± SD (Median)	12.16 ± 6.05 (12)	2.21 ± 1.67 (2)		

Data are presented as Minimum–Maximum and Mean±Standard Deviation (Median).

Mann-Whitney U Test was used.

**p < 0.01.

**Table 3:** Evaluation of panic disorder severity scale (PDSS) scores in the patient and control groups.

	Patient (*n* = 81)	Control (*n* = 81)	Test Value	p-value
Min–Max	8–34	0–2	**41.00**	< 0.001**
Mean ± SD (Median)	18.44 ± 5.02 (19)	0.21 ± 0.47 (0)		

Data are presented as Minimum–Maximum and Mean±Standard Deviation (Median).

Mann-Whitney U Test was used.

**p < 0.01.

Table 2. Pittsburgh Sleep Quality Index (PSQI) Scores in the Patient and Control Groups.

Table 3. Montgomery–Åsberg Depression Rating Scale (MADRS) Scores in the Patient and Control Groups.

**Table 4:** Evaluation of Montgomery–Åsberg depression rating scale (MADRS) scores in the patient and control Groups.

	Patient (*n* = 81)	Control (*n* = 81)	Test Value	p-value
**Min–Max**	2–37	0–5	**124.50**	< 0.001**
**Mean ± SD (Median)**	12.16 ± 6.05 (12)	2.21 ± 1.67 (2)		

Data are presented as Minimum–Maximum and Mean±Standard Deviation (Median).

Mann-Whitney U Test was used.

**p < 0.01.

Table 4. Panic Disorder Severity Scale (PDSS) Scores in the Patient and Control Groups.

### Neuropsychological performance – group differences

Neuropsychological Performance – Group Differences WCST results indicated that patients with PD had significantly lower total correct responses (85.15 ± 19.71; *p* < 0.001) compared to healthy controls. They also completed fewer categories and demonstrated lower percentages of conceptually correct responses (57.57% vs. 71.26%; *p* < 0.001). In contrast, patients showed higher total error rates, including both perseverative (23.36 ± 14.48; *p* < 0.001) and non-perseverative errors. IGT performance was similarly impaired in the PD group, with significantly lower net scores (mean: − 8.73) compared to the control group (1.96; *p* = 0.007) (see Tables 5 and 6).

**Table 5:** Evaluation of Wisconsin card sorting test (WCST) results in the patient and control group.

WCST variables	Patient (*n* = 81)	Control (*n* = 81)	Test Value	*p*-value
Total number of correct responses	85.15 ± 19.71 (90)	98.46 ± 13.62 (102)	1789.00	< 0.001**
Total number of errors	42.43 ± 19.73 (37)	29.54 ± 13.62 (26)	1836.00	< 0.001**
Total number of perseverative responses	27.01 ± 18.95 (21)	18.28 ± 14.76 (13)	1986.00	< 0.001**
Total number of non-perseverative errors	18.83 ± 10.97 (17)	13.32 ± 6.26 (13)	2112.50	< 0.001**
Total number of perseverative errors	23.36 ± 14.48 (19)	16.22 ± 11.14 (13)	1904.50	< 0.001**
Total number of categories	4.75 ± 2.96 (4)	6.83 ± 2.48 (8)	1967.00	< 0.001**
Perseverative error percentage	18.42 ± 11.44 (15)	12.74 ± 8.74 (10.16)	1852.50	< 0.001**
Number of responses to complete first category	24.87 ± 19.66 (16)	17.23 ± 10.76 (12)	2157.50	0.002**
Conceptual level response percentage	57.57 ± 20.86 (63)	71.26 ± 15.02 (75)	1813.00	< 0.001**
Failure to maintain set	1.70 ± 1.62 (1)	1.74 ± 1.60 (1)	3199.00	0.779
Learning to learn score	−1.65 ± 7.09 (0.11)	−0.41 ± 3.87 (0.05)	2477.00	0.814

Data are presented as Mean±Standard Deviation (Median).

Mann-Whitney U Test was used.

**p < 0.01.

**Table 6:** Evaluation of Iowa gambling task (IGT) results in the patient and control groups.

	Patient (*n* = 81)	Control (*n* = 81)	Test Value	p-value
Min–Max	−55-30	[−40]−56	**2238.50**	< 0.001**
Mean ± SD (Median)	−7,86 ± 14,67 (−6)	0,64 ± 13,27 (0)		

Data are presented as Minimum–Maximum and Mean±Standard Deviation (Median).

Mann-Whitney U Test was used.

**p < 0.01.

Table 5. Wisconsin Card Sorting Test (WCST) Performance in the Patient and Control Groups.

Table 6. Iowa Gambling Task (IGT) Performance in the Patient and Control Groups.

### Clinical and cognitive ımprovement after treatment

Among the 38 patients who completed the three-month follow-up, significant improvements were observed in both clinical and cognitive measures (see Table 7). Clinically, PSQI scores decreased from 10.08 to 4.37, MADRS scores from 12.4 to 4.71, and PDSS scores from 18.55 to 4.03 (all *p* < 0.001). Cognitively, patients showed increased total correct responses on the WCST (86.75 → 99.97), reduced perseverative errors (21.83 → 14.53; *p* = 0.001), and improved percentages of conceptually correct responses (59.98% → 72.73%; *p* = 0.002). IGT performance also improved, with net scores increasing from − 8.73 to 0.11 (*p* = 0.023).

**Table 7:** Evaluation of changes in PSQI, MADRS, PDSS, WCST, and IGT scores before and after treatment in the patient group (*n* = 38).

Measure	Pre-treatment Mean ± SD (Median)	Post-treatment Mean ± SD (Median)	Test statistic†	*p*-value
PSQI	10.08 ± 3.07 (10)	4.37 ± 1.89 (4)	−5.169	< 0.001**
MADRS	12.4 ± 6.82 (11)	4.71 ± 3.93 (3)	−4.568	< 0.001**
PDSS	18.55 ± 4.95 (19)	4.03 ± 1.75 (4)	−5.376	< 0.001**
Total correct responses	86.75 ± 17.7 (90.5)	99.97 ± 13.27 (104)	−3.546	< 0.001**
Total errors	40.40 ± 17.66 (36)	28.03 ± 13.27 (24)	−3.508	< 0.001**
Total perseverative responses	24.95 ± 13.55 (21)	15.92 ± 10.25 (12)	−3.260	0.001**
Total non-perseverative errors	18.08 ± 10.17 (17)	13.50 ± 6.11 (13.5)	−2.446	0.014*
Total perseverative errors	21.83 ± 10.5 (20)	14.53 ± 8.32 (11.5)	−3.323	0.001**
Total number of categories	4.85 ± 2.96 (4)	6.42 ± 2.63 (6)	−2.195	0.028*
Perseverative error percentage	17.22 ± 8.33 (15.63)	11.35 ± 6.5 (8.99)	−3.199	0.001**
Number of responses to complete first category	25.26 ± 23.23 (14)	20.13 ± 13.4 (14)	−0.795	0.427
Conceptual level response percentage	59.98 ± 19.38 (66)	72.73 ± 14.41 (76)	−3.078	0.002**
Failure to maintain set	1.80 ± 1.54 (1)	2.42 ± 2.04 (2)	−1.677	0.094
Learning to learn score	−2.59 ± 7.9 (0.105)	−1.16 ± 7.26 (0.26)	−0.973	0.331
IGT	−8.73 ± 15.87 (−8)	0.11 ± 19.68 (0)	−2.280	0.023*

Data are presented as Mean±Standard Deviation (Median).

Wilcoxon Signed-Rank Test was used.

*p < 0.05, **p < 0.01.

Table 7. Changes in PSQI, MADRS, PDSS, WCST, and IGT Scores in the Patient Group Before and After Treatment (*n* = 38).

### Correlations between clinical and cognitive variables

Improvement in sleep quality (PSQI) was strongly correlated with reductions in depressive (MADRS) and panic (PDSS) symptom severity (*r* = 0.581, *p* < 0.001 and *r* = 0.473, *p* = 0.003, respectively). A similarly strong correlation was found between MADRS and PDSS scores (*r* = 0.606, *p* < 0.001). Although changes in PSQI scores were not significantly correlated with WCST subdimensions, the change in IGT performance was significantly associated with improvements in PDSS scores (*r* = 0.327, *p* = 0.045). Further details are provided in Supplementary Tables 1 and 2.

### GEE analysis – relationship between sleep quality and decision-making ability

Sleep Quality and Decision-Making GEE analysis showed that improvements in sleep quality were significantly associated with better decision-making performance. Specifically, each one-point decrease in PSQI was associated with a 0.156-point increase in IGT score (β = − 0.156; SE = 0.062; Wald χ² = 6.162; *p* = 0.013). The time variable (baseline vs. follow-up) was not a significant predictor in the model (*p* = 0.386). These findings suggest that improved sleep quality during treatment was independently associated with higher IGT performance in patients with PD (see Table 8).

**Table 8:** Evaluation of the effect of PSQI scores on post-treatment IGT scores using the GEE model.

Parameters	B	SH	%95 Wald CL	Wald Chi-Square	df	*p*-value
*Intercept*	3.235	0.477	[2.301–4.169]	46.087	1	< 0.001**
*Time*	0.429	0.495	[− 0.541–1.398]	0.751	1	0.386
*PSQI*	−0.156	0.062	[− 0.278–−0.033]	6.162	1	0.013*

Dependent variable: IGT Score.

SE: Standard Error; CI: Confidence Interval; df: Degrees of Freedom.

*p < 0.05, **p < 0.01.

Table 8. The Effect of Post-Treatment PSQI Scores on IGT Performance: Results of the GEE Model.

## Discussion

This study indicates that improvements in sleep quality are associated with cognitive improvements. Meta-analytic evidence suggests that insomnia substantially impairs cognitive performance, whereas better sleep quality is associated with improved cognitive outcomes^34^. In the context of PD, enhancing sleep quality may be associated with fewer cognitive deficits, particularly in executive functioning and decision-making abilities.

The WCST is a widely used neuropsychological instrument for assessing executive functions. A high number of perseverative errors in the WCST indicates difficulty in shifting from a previously valid sorting rule to a new one, reflecting impaired cognitive flexibility. In contrast, non-perseverative errors—such as incorrect category choices—may signal deficits in attention, planning, or rule comprehension. The number of categories completed reflects the ability to detect and consistently apply correct classification strategies, while set maintenance performance reflects sustained adherence to a given rule^35,36^. Previous research has shown that individuals with PD often exhibit impairments in executive functioning. For instance, patients with PD have been shown to have significantly higher rates of perseverative errors than healthy controls when assessed using the WCST. Additionally, these patients demonstrated significantly lower performance in set maintenance and in the percentage of conceptually correct responses^20^. However, findings regarding executive functioning in PD remain somewhat inconsistent. Recent reviews suggest that while global executive dysfunction may not be present in most patients, specific impairments—particularly in attention, cognitive flexibility, decision-making, and working memory—are frequently observed^12^.

Performance on the WCST was significantly poorer in the PD group compared to the healthy control group. Patients exhibited a significantly lower total number of correct responses, completed fewer categories, and had a reduced percentage of correct answers at the conceptual level. In contrast, the total number of incorrect responses, perseverative errors, and non-perseverative errors were significantly higher in the patient group.

However, no significant group differences were observed in certain subcomponents, such as the learning-to-learn score and set maintenance performance. This suggests that some aspects of executive functioning may remain relatively preserved in individuals with PD. According to the executive function model, executive functions consist of separable but interrelated components, each of which may be differentially affected^37^. The current findings suggest that structured and stable cognitive processes, particularly those related to rule maintenance, may be less impaired in individuals with PD. Supporting this, meta-analytic evidence indicates that stress significantly impairs cognitive flexibility and working memory, while certain executive abilities appear more resilient^38^. These findings reinforce the notion that executive dysfunction in PD may be selective rather than global in nature^12^.

Decision-making refers to the cognitive process by which an individual selects a course of action from among available alternatives^39^. Neuroimaging studies suggest that heightened amygdala activation and prefrontal cortex dysfunction in individuals with PD contribute to increased sensitivity to threat and a tendency to avoid potential losses in uncertain situations^40^. This heightened sensitivity may impair risk evaluation in individuals with elevated anxiety.

Research indicates that loss aversion is more pronounced in anxious individuals, possibly due to disrupted connectivity between the amygdala and prefrontal regulatory regions^41–44^. Such disruptions may underlie compromised decision-making abilities observed in PD. In our study, participants with PD performed significantly worse on the Iowa Gambling Test (IGT) compared to healthy controls. This result is consistent with previous research showing that elevated anxiety adversely affects decision-making performance.

A meta-analysis of anxiety disorders found that hypersensitivity to threatening stimuli and a tendency to avoid negative outcomes contribute to impaired decision-making^45^. Furthermore, individuals with high trait anxiety have been shown to perform poorly on the IGT, a deficit associated with increased physiological arousal and cognitive avoidance tendencies^46,47^. A recent review also noted that patients with PD may display deficits in executive function and disturbances in complex cognitive operations, including decision-making under uncertainty^12^.

Our findings suggest that cognitive impairments in PD extend beyond executive dysfunction and include deficits in decision-making processes.

Additionally, we observed a negative correlation between the severity of PD symptoms and sleep quality; however, this association did not reach statistical significance (*r* = − 0.21, *p* = 0.079). This may be due to the limited sample size. Nonetheless, the trend points to a possible relationship between poorer sleep quality and greater panic symptom severity. Previous literature has also suggested a potential link between sleep disturbances and increased panic symptoms^48^. Future studies with larger samples are warranted to more accurately evaluate this relationship.

The relationship between sleep quality and cognitive functioning in our sample revealed a significant association between PSQI scores and several subcomponents of the WCST. Higher PSQI scores—reflecting poorer sleep quality—were associated with a lower number of correct responses, fewer completed categories, and reduced percentages of conceptual-level responses. These findings suggest that impaired sleep may be linked to greater deficits in executive functions, including cognitive flexibility, abstraction, and problem-solving abilities.

A recent meta-analysis reported that individuals with chronic insomnia show significant impairments in cognitive domains such as attention, working memory, and problem-solving^34^. Therefore, some executive function impairments observed in patients with PD may be partially attributable to poor sleep quality.

In our study, treatment was found to improve both clinical symptoms and cognitive performance. Of the 81 patients, 38 underwent follow-up assessment at the end of the 3-month treatment period, and detailed analyses were conducted on this subgroup. Post-treatment evaluations revealed significant reductions in PSQI, MADRS, and PDSS scores compared to baseline (*p* < 0.01 for all). These results show reductions in panic symptoms, depressive symptoms, and PSQI scores over the course of treatment.

In terms of cognitive outcomes, patients showed notable post-treatment improvements on the WCST. There were significant increases in total correct responses, number of completed categories, and percentages of conceptual-level responses (*p* < 0.01), reflecting enhanced problem-solving skills and the ability to learn and apply new rules. Additionally, significant reductions were observed in total incorrect responses, number and percentage of perseverative errors, and non-perseverative errors (*p* < 0.05), suggesting improvements in attention, processing speed, and response inhibition.

Previous longitudinal research in adolescents found that total error rates and non-perseverative errors were associated with mood and anxiety symptoms, whereas perseverative errors were not significantly linked to these symptoms^49^. In contrast, our findings demonstrated a significant reduction in perseverative errors, suggesting a pattern consistent with improved cognitive flexibility over time. This discrepancy may be due to differences in age group, clinical characteristics, or treatment approach.

Our study demonstrated a significant improvement in Iowa Gambling Test (IGT) scores following treatment compared to pre-treatment levels (*p* < 0.05). This finding suggests a shift toward less risky decision-making and greater attention to long-term outcomes over time.

Previous research has shown that decision-making processes are often impaired in individuals with PD. Patients with PD have been reported to perform worse on the IGT than healthy controls, a deficit attributed to heightened sensitivity to bodily sensations, which negatively influences their decision-making^50^. The same study suggested that treatments targeting core symptoms may help mitigate these cognitive impairments.

In our sample, post-treatment improvements were reflected in an increased rate of advantageous choices and a stronger inclination to avoid disadvantageous options. Prior literature supports the notion that improved sleep quality positively influences decision-making performance. Previous studies have shown that participants who obtained a full night’s sleep selected more advantageous cards on the IGT and learned the game rules more quickly than sleep-deprived participants^51^. Similarly, individuals who slept after learning demonstrated a reduced preference for the disadvantageous “B” deck and showed more strategic decision-making^52^.

Recent studies have reinforced these findings. For instance, IGT performance among nurses was significantly impaired after night shifts with inadequate rest, and poor sleep was associated with a greater tendency toward risky choices^53^. Similarly, patients with primary insomnia selected disadvantageous decks more frequently than healthy controls, highlighting the adverse effects of sleep disturbance on decision-making^54^.

In line with this literature, our findings suggest that improved sleep quality is associated with better decision-making performance in patients with PD. Specifically, enhanced sleep appears to support a reduced preference for risky options and a greater capacity to prioritize long-term benefits^54^.

The results of the GEE analysis conducted in this study indicate that improvements in sleep quality during treatment are significantly associated with better decision-making performance, independent of reductions in depression or anxiety symptoms. Even after controlling for scores on the MADRS and PDSS, each one-point improvement in PSQI was associated with a 0.156-point increase in IGT performance (β = 0.156, *p* = 0.013). This suggests that sleep quality shows an independent association with decision-making performance.

The concurrent improvements in symptom severity and sleep quality appear to make distinct and clinically meaningful contributions to the recovery of cognitive dysfunction in PD. This finding is consistent with previous literature showing that the treatment of insomnia can enhance sleep quality, cognitive performance, and even promote neuroplasticity^55^.

In this context, sleep-focused interventions should be considered an essential component of PD treatment, rather than limiting care to anxiety symptom reduction alone. Existing literature often treats sleep disturbances and cognitive impairments in PD as separate issues. However, this study demonstrates that sleep quality is closely associated with executive functioning and decision-making supporting a more integrated and sleep-informed treatment approach.

It is important to note that the exclusion of sleep disturbance from the diagnostic criteria for PD in the DSM-5 may contribute to its frequent underrecognition by clinicians^3^. While insomnia is highly prevalent across anxiety disorders—affecting approximately 70–80% of individuals—and is explicitly included in diagnostic criteria for conditions such as generalized anxiety disorder and post-traumatic stress disorder, its omission in the criteria for PD may lead to gaps in clinical assessment^55^.

The findings of this study highlight that improved sleep quality appears to be closely related to cognitive improvements observed in PD. Therefore, evaluating sleep patterns and complaints should be a standard part of the mental status examination in patients with PD. Addressing sleep disturbances may be an important component of clinical management.

Enhancing sleep quality not only supports cognitive functioning but also contributes to overall daily functioning. Such improvements are likely to enhance both patients’ quality of life and the overall effectiveness of treatment^48^. Recent studies further suggest that treating insomnia can positively affect the course of various psychiatric conditions, with cognitive behavioral therapy for insomnia (CBT-I) showing particular promise for anxiety disorders^56,57^.

In summary, our findings underscore the importance of incorporating sleep-focused interventions into the treatment of PD. This approach may help alleviate cognitive impairments and support sustained improvements in both treatment outcomes and long-term quality of life.

## Limitations

This study has several limitations that should be acknowledged. First, the follow-up analyses were conducted in a subset of patients who completed the three-month assessment, which may have introduced attrition bias and limited the generalizability of the findings. Control participants were not reassessed at follow-up because the study was designed to examine within-patient clinical and cognitive changes during routine care; controls were not receiving treatment or clinical monitoring, and repeated testing would primarily introduce practice effects rather than clinically meaningful change. Baseline sleep quality, depressive and panic symptom severity, and cognitive performance did not differ between patients who completed the follow-up and those who did not (all *p* > 0.20), reducing the likelihood of systematic attrition bias; however, the reduced sample size still limits the generalizability of the longitudinal findings. Second, the naturalistic design did not allow for control over pharmacological treatments or psychotherapy involvement; variations in medication type, dose, duration, or concurrent psychological interventions may therefore have influenced clinical and cognitive outcomes. Third, a subset of control participants were hospital employees with occasional night duties. Although these shifts were infrequent, typically allowed partial sleep, and sensitivity analyses excluding these individuals did not change the magnitude or significance of the group differences, residual confounding related to occupational or circadian factors cannot be fully dismissed. Moreover, the follow-up sample size (*n* = 38) did not provide sufficient statistical power to perform reliable subgroup comparisons between clinical or sleep ‘improvers’ and ‘non-improvers’, and such analyses were therefore not conducted.

Fourth, cognitive performance was assessed using only two tasks (WCST and IGT). While both are well-validated, they may not capture the full spectrum of executive functioning and decision-making processes. Fifth, sleep quality was measured solely through a self-report questionnaire (PSQI), which may be subject to reporting biases and may not fully reflect objective sleep disturbances. Additionally, two patients had stable, previously diagnosed primary sleep disorders (one OSA and one RLS). Excluding these individuals did not alter the results, but subtle residual confounding related to subclinical sleep disturbances remains possible.

Finally, although detailed information on antidepressant, benzodiazepine, and other sedative medication use was provided, and pharmacotherapy followed routine clinical practice rather than a standardized protocol, the potential influence of these agents on sleep quality and cognitive performance cannot be entirely ruled out. Given the number of statistical comparisons conducted, the absence of formal correction for multiple testing may increase the risk of Type I error, and the findings should be interpreted with appropriate caution.

Future research should aim to replicate these findings in larger and more diverse samples, incorporate objective sleep assessments such as actigraphy or polysomnography, and more rigorously control for treatment-related variables. Including a broader range of cognitive domains and employing experimental or longitudinal intervention designs may further clarify the causal pathways linking sleep and cognition in panic disorder.

## Conclusion

This study provides novel evidence that improvements in sleep quality during routine treatment are associated with cognitive gains—particularly in decision-making capacity—among individuals with PD. While PD has traditionally been conceptualized and treated primarily as an episodic anxiety condition, our findings highlight the broader cognitive consequences of the disorder and the therapeutic relevance of addressing sleep disturbances. Notably, the association between sleep improvement and IGT performance persisted even after controlling for changes in depressive and anxiety symptoms.

Although executive function (as measured by the WCST) showed partial improvement over the three-month follow-up, the observed trend toward better cognitive flexibility and reduced perseverative errors underscores the potential relevance of naturalistic sleep recovery to broader aspects of cognitive control. These findings emphasize the importance of integrating sleep assessment and management into routine psychiatric evaluation and intervention strategies for PD.

Nevertheless, the observational and naturalistic design, reliance on self-reported sleep measures, and limited follow-up sample size warrant caution in interpreting the findings. Future studies incorporating objective sleep assessments and experimental designs could further clarify the mechanisms linking sleep and cognition. Overall, our results suggest that sleep quality is not merely a secondary concern but a potentially modifiable factor associated with cognitive recovery and improved functional outcomes in PD. Addressing sleep disturbances may enhance both clinical remission and cognitive resilience, thereby enriching the scope and efficacy of treatment.

## Supplementary Information

Below is the link to the electronic supplementary material.

Supplementary Material 1

Supplementary Material 2

## Data Availability

The datasets generated and/or analyzed during the current study are available from the corresponding author on reasonable request.
