# Supplementary material for: Improved sleep quality is independently associated with decision-making recovery in panic disorder: a longitudinal study
Source: Sci Rep. 2026 Feb 4;16:7201. doi: 10.1038/s41598-026-37946-5 (PMC12923609; doi:10.1038/s41598-026-37946-5)
Supplement: Supplementary file 1 — Supplementary Material 1 [file 41598_2026_37946_MOESM1_ESM.docx]

**Supplementary Table 1. Correlation Analysis of the Differences in PSQI, MADRS, PDSS, WCST, and IGT Scores Before and After Treatment in the Patient Group (n=38)**

|  | Difference in PSQI | | Difference in MADRS | | Difference in PDSS | |
| --- | --- | --- | --- | --- | --- | --- |
|  | **r** | **p** | **r** | **p** | **r** | **p** |
| **Total number of correct responses** | 0.051 | 0.764 | 0.092 | 0.582 | -0.146 | 0.382 |
| **Total number of errors** | -0.095 | 0.575 | -0.140 | 0.403 | 0.094 | 0.573 |
| **Total number of perseverative responses** | 0.156 | 0.358 | -0.047 | 0.777 | 0.178 | 0.284 |
| **Total number of non-perseverative errors** | -0.113 | 0.506 | -0.009 | 0.957 | 0.200 | 0.228 |
| **Total number of perseverative errors** | 0.146 | 0.387 | -0.079 | 0.638 | 0.188 | 0.259 |
| **Total number of categories** | 0.075 | 0.659 | 0.061 | 0.715 | -0.144 | 0.387 |
| **Perseverative error percentage** | 0.140 | 0.415 | -0.120 | 0.480 | 0.166 | 0.325 |
| **Number of responses to complete first category** | -0.230 | 0.177 | -0.205 | 0.223 | 0.006 | 0.971 |
| **Conceptual level response percentage** | 0.089 | 0.605 | 0.120 | 0.478 | -0.143 | 0.398 |
| **Failure to maintain set** | -0.118 | 0.488 | -0.002 | 0.992 | 0.094 | 0.576 |
| **Learning to learn score** | -0.224 | 0.251 | 0.065 | 0.738 | 0.098 | 0.614 |
| **IGT** | 0.135 | 0.425 | 0.154 | 0.356 | 0.327 | 0.045* |

*Spearman Correlation Coefficient was used.*

*r: correlation coefficient; p: p-value.*

**p<0.05*
