# Supplementary material for: Improved sleep quality is independently associated with decision-making recovery in panic disorder: a longitudinal study
Source: Sci Rep. 2026 Feb 4;16:7201. doi: 10.1038/s41598-026-37946-5 (PMC12923609; doi:10.1038/s41598-026-37946-5)
Supplement: Supplementary file 2 — Supplementary Material 2 [file 41598_2026_37946_MOESM2_ESM.docx]

**Supplementary Table 2. Correlation Analysis of the Differences in PSQI, MADRS, and PDSS Scores Before and After Treatment in the Patient Group (n=38)**

| **Variables** | **r** | **p-value** |
| --- | --- | --- |
| **Difference in PSQI–MADRS** | 0.581 | <0.001** |
| **Difference in PSQI–PDSS** | 0.473 | 0.003** |
| **Difference in MADRS–PDSS** | 0.606 | <0.001** |

*Spearman Correlation Coefficient was used.*

***p<0.01*
